# Organoclay-assisted disperse dyeing of polypropylene nanocomposite fabrics in supercritical carbon dioxide

**DOI:** 10.1038/s41598-024-63192-8

**Published:** 2024-06-12

**Authors:** Tarek A. Elmaaty, Abdalla A. Mousa, Reham Farouk, Hanan Elsisi, Heba Sorour, Yehya A. Youssef, Dina Abbas

**Affiliations:** 1 Department of Textile Printing, Dyeing and Finishing, Faculty of Applied Arts, Damietta University, Damietta, 34512 Egypt; 2 Dyeing, Printing and Textile Auxiliaries Department, National Research Centre, Textile Research and Technology Institute, 33 EL Buhouth St., Dokki, Giza, 12622 Egypt

**Keywords:** Chemistry, Green chemistry

## Abstract

Dyeing using supercritical carbon dioxide (SC-CO_2_) is carried out on the polypropylene (PP) nanocomposite fabrics with model disperse dye compared with their regular fabric at various dyeing temperatures and pressures. The results are compared with those obtained in aqueous dyeing method. The PP nanocompsite fabrics dyed in SC-CO_2_ medium exhibited higher colour strength (K/S) values compared with their PP regular fabric. The PP nanocompsite fabrics and their regular fabric dyed in SC-CO_2_ medium have higher K/S values than those dyed in aqueous medium. The color coordinates of all PP fabrics dyed in SC-CO_2_ and aqueous medium were positive with respect to a* and b* coordinates depending on the disperse red dye uptake. The PP nanocomposite fabrics dyed in SC-CO_2_ and aqueous medium exhibited higher antibacterial properties than their regular fabrics. All PP fabrics dyed in SC-CO_2_ and aqueous medium present very good washing, perspiration and light fastness.

## Introduction

PP fabric has attractive properties, such as low specific gravity, good antistatic character and good chemical resistance, as well as wide availability and low cost. These properties make PP fabric a versatile and useful fabric in the manufacture of home furnishings and industrial applications^1^. PP fabric does not include a functional group that is effective for chemical reactions with dyes. PP fabric also has a relatively dense crystalline structure, extremely high hydrophobicity and is hardly swollen by water. For these reasons, the dyeing of PP fabric using aqueous medium is considered to be difficult. Several approaches, including PP fiber modifications via plasma and ultrasonication treatments, as well as the use of new colourants and nanoparticles (as example nanoclays) have been extensively explored to upgrade the structural and functional properties of PP fabrics and improve their dyeability^2–7^. Polymer–clay nanocomposites have garnered a lot of attention due to their potential for improved mechanical, thermal, barrier, and physical properties in comparison to other kinds of composite materials. So, the applications of nanoclay, have been the subject of recent research work on the synthetic fabrics. Organoclay-assisted vat and disperse dyeing of synthetic PET (polyethylene terephthalate) nanocomposite fabric by melt spinning have recently been reported. The prepared PET nanocomposite fabrics using different montmorillonite (MMT) organic modified clays exhibited higher K/S using vat and disperse dyes compared with those of the reference fabrics made from fibres spun without MMT clay content and regular fabrics^8^. Also organoclay-assisted vat dyeing of PP nanocomposite fabrics have recently been reported. The prepared PP nanocomposite fabrics containing MMT exhibited higher colour strength using vat dyes compared to reference fabrics made from fibres spun without MMT clay content^9^. Continuity to the developments of improving the dyeability of the synthetic fabrics, SC-CO_2_ is the perfect replacement for aqueous medium to overcome the problems of conventional dyeing. Dyeing in SC-CO_2_ is based on replacing water with CO_2_ at its critical point under high temperature and pressure^10–21^. Various dyeing processes and approaches have been developed that employ SC-CO_2_ for coloration of PP fabrics^22–28^. A semi-continuous process using SC-CO_2_ is reported for preparation of PP nanocomposite materials using surface modified MMT^29^. To the best of our knowledge, no work has been published concerning the dyeing of PP nanocomposite fabrics in SC-CO_2_ medium using disperse dyes. In a continuation of our previous work^9^, the aim of the current study is to demonstrate the possibility of dyeing PP nanocomposite fabrics with model disperse dye in SC-CO_2_ medium. This paper will describe the effect of intercalating agents, as a modifier for MMT, on the disperse dyeing properties of the resultant PP/MMT nanocomposite fabrics in SC-CO_2_ medium.

## Materials and methods

### Organoclays

Three commercial types of modified MMT were purchased from Sigma-Aldrich (MMT-A, B, C) and their specifications are listed in Table 1.

**Table 1:** Overview of the different nanoclays used.

Modified MMT clay	Modifier name	Place of modification	Modifier chemical structure
682632 Aldrich **(MMT-A)**	35 wt% octadecylamine 0.5 -5 wt% aminopropyltriethoxy silane	Interlayer surfaces and edges	
682608 Aldrich **(MMT-B)**	25–30 wt% Trimethylstearyl ammonium chloride	Interlayer	
682616 Aldrich **(MMT-C)**	35 wt% octadecylamine	Interlayer	

### Polymers

PP granules (Moplen HP561R) were provided by Moplen.

### Regular PP fabric

Regular PP fabric (135 g/m^2^, yarn count: dtex 233/32) was kindly received from DITF, Denkendorf, Germany and treated before dyeing with an aqueous solution containing 2 g/L nonionic detergent (Sera Wash M-RK, DyStar, Egypt) at a liquor to goods ratio 50:1 and at 80 °C for 30 min, and then rinsed and allowed to dry in the open air.

### Preparation of PP nanocomposite fabrics

PP nanocomposite fabrics prepared according to our previous work. Spinnable PP polymers were obtained by mixing PP master batches with 4 wt% MMT, possessing either hydrogenated amine organomodified nanoclays (MMT-A and C) or quaternary ammonium-based organomodified nanoclay (MMT-B) intercalating compounds with a pure untreated PP to MMT content of 0.5 wt%. After spinning, the prepared nanocomposites were drawn and knitted into fabrics^9^.

### Preparation of model disperse dye

**Figure 1:**
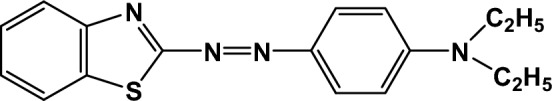
2-((4’-N,N-diethylaminophenyl)azo) benzothiazole “Disperse Red Dye”.

The disperse red 2-((4’-N,N-diethylaminophenyl)azo) benzothiazole dye, shown in Fig. 1 was designed for use in SC-CO_2_ and synthesized by the method previously described in literature ^30^.

### SC-CO_2_ dyeing of PP nanocomposite fabrics and their regular fabric with disperse red dye

**Figure 2:**
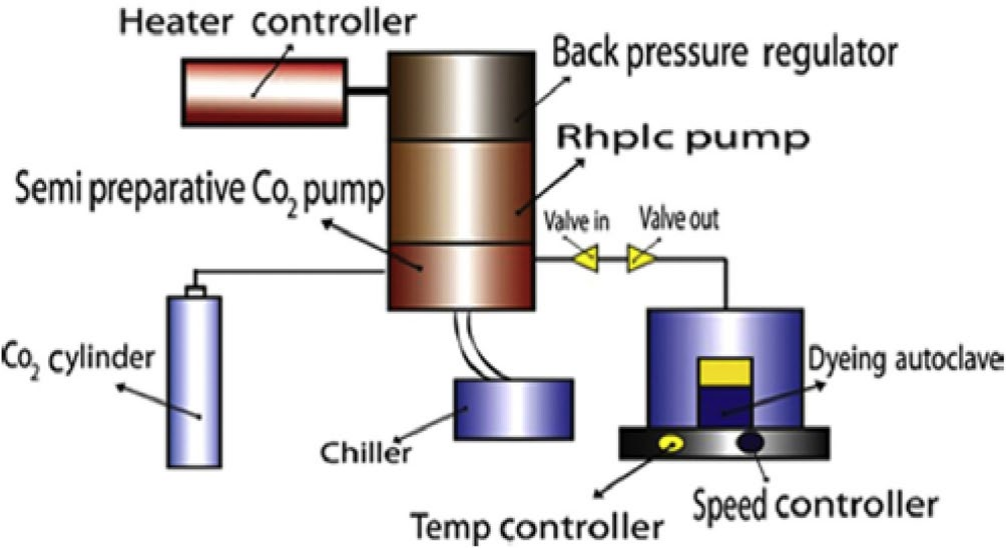
A diagram of the lab-scale SC-CO_2_ dyeing apparatus.

SC-CO_2_ apparatus consists of the following main parts as shown in Fig. 2: a cylinder of carbon dioxide, a chiller (model Julabo FL601), a semi-preparative CO_2_ pump (model JASCOPU-4386), an RHPLC pump for extraction (model JASCO PU-4180), a back pressure regulator (model JASCO BP-4340) which has a maximum rate of pressure at 300 bar, a heater controller (model HC-2068-01), a dyeing autoclave, a temperature controller and speed controller (model EYELARCX-1000 H) which has a maximum rate of temperature at 130 °C, an internal capacity approximately 50 ml, a maximum CO_2_ flow rate of a circulation pump 10 mL/min, however, can reach 30 mL/min at optional heater”^11^.

The prepared disperse dye (3% shade without dispersing agent) was placed inside the SC-CO_2_ dyeing vessel. PP nanocomposite fabrics (MMT-A, B and C) and their regular fabric were placed also in the SC-CO_2_ dyeing vessel. The CO_2_ fluid preheated, and then charged into the dyeing vessel. At both temperature and pressure required, the dyeing is completed at the dyeing time 1 h. Finally, decreasing both temperature and pressure and then the dyeing vessel was opened and the dyed PP nanocomposite fabrics and their regular fabric were then rinsed with cold water and air dried for colour measurements, color fastness and antibacterial activity.

### Aqueous dyeing of PP nanocomposite fabrics and their regular fabric with disperse red dye

**Figure 3:**
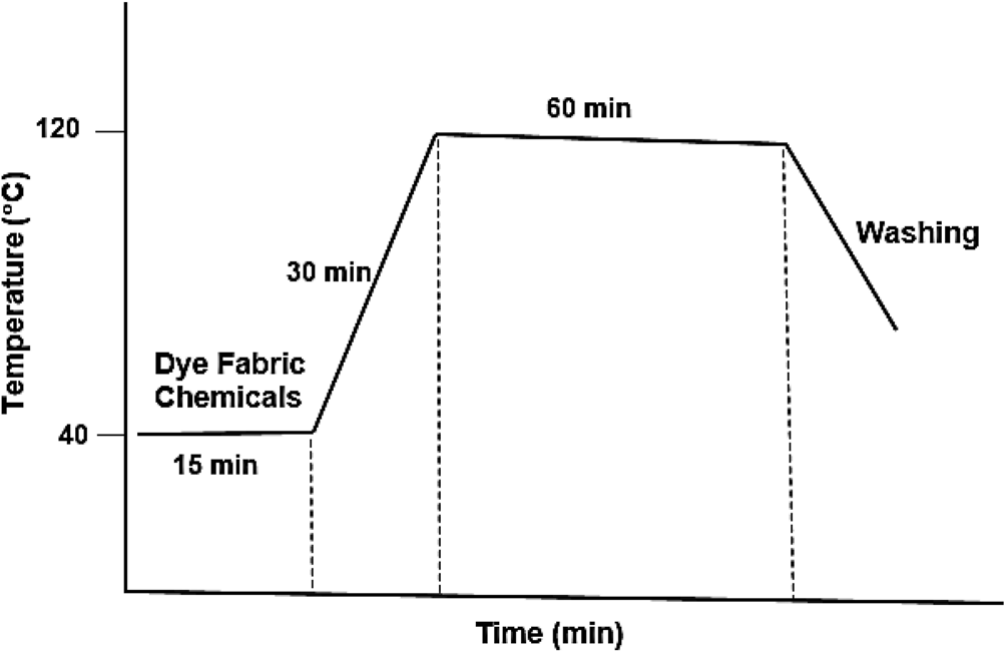
Aqueous dyeing curve.

Aqueous dyeing was carried out on a Pyrotec infrared laboratory dyeing unit (Roaches International). PP nanocompoisite fabrics and regular fabric were introduced into a dye bath containing 3% owf of disperse red dye concentration, 2 g/L dispersing agent at 40 °C and at a liquor ratio of 100:1. The dye bath pH was adjusted to 4–5, and then the temperature was raised to 120 °C, over 30 min, and maintained at this temperature for 60 min. The dyed fabrics were then rinsed with cold water and air dried (Fig. 3).

### Color measurements

The K/S values and color coordinates (L*, a*, b*, C* and h°) of dyed PP nanocompoisite fabrics and their regular fabric were measured using an UltraScan PRO spectrophotometer (HunterLab, USA). L* represents the lightness coordinate. a* represents the red/green coordinate, with + a* indicating red, and − a* indicating green. b* represents the yellow/blue coordinate, with + b* indicating yellow, and − b* indicating blue. C* represents the chroma coordinate. *h*° represents the hue angle, expressed in degrees, with 0° being a location on the + a* axis (red), continuing to 90° for the + b* axis (yellow), 180° for—a* (green), 270° for—b* (blue), and back to 360° = 0°.

The levelling of the dyed PP samples in SC-CO_2_ and aqueous medium was evaluated by measuring the colour differences within each sample at five separate points and the average colour difference (∆E) between these points was determined ^31,32^.

### Antibacterial activity

The antibacterial behavior of dyed PP nanocomposite fabrics and their regular fabric was evaluated against two bacterial strains; gram-negative *Escherichia coli* (G −) and gram-positive *Staphylococcus aureus* (G +) according to *ASTM E2149-10* using Colony Counting Method ^33^.

### Fastness testing

The color fastness of the dyed PP nanocompoisite fabrics and their regular fabric in SC-CO_2_ and aqueous medium were assessed according to ISO standard methods^34–37^. Fastness to washing was carried out according to *ISO 105-C06 B2S*. Fastness to an acidic and alkaline perspiration was carried out according to *ISO 105-E04*. The colour changing of PP fabrics and colour staining of the adjacent multi-fiber were then assessed with the ISO grey scales. Light fastness was also assessed according to *ISO105-B02* using a Xenon arc lamp test.

## Results and discussion

### SC-CO_2_ disperse dyeing of PP nanocomposite fabrics and their regular fabric

**Figure 4:**
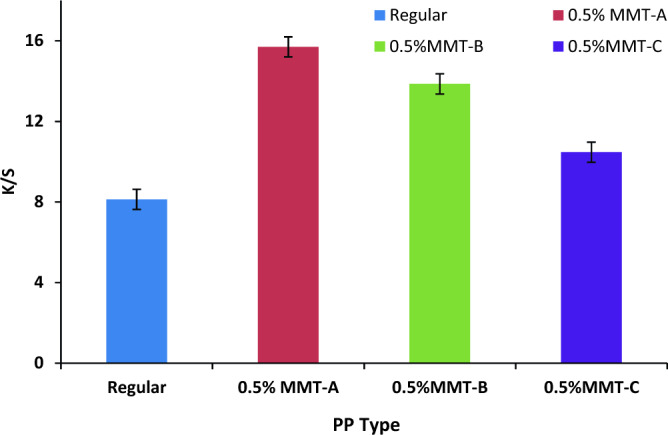
The K/S values of PP nanocomposite fabrics and their regular fabric dyed in SC-CO_2_ medium with model disperse dye.

The SC-CO_2_ disperse dyeing behaviour of PP nanocomposite fabrics containing various types of organoclay MMT (MMT-A, B and C) was compared with PP regular fabric using model disperse dye at (250 bar, 120 °C, 3% shade and 1 h). The results in Fig. 4 show that the prepared PP nanocomposite fabrics containing hydrogenated amine organomodified nanoclays (MMT-A and C) or quaternary ammonium-based organomodified nanoclay (MMT-B) exhibited higher K/S values using model disperse dye compared with PP regular fabric. The low K/S value of the PP regular fabric was anticipated because of the lack of dye sites, low water sorption and the highly hydrophobic character. This is in agreement with that the dyeing behavior of nanocomposite fabrics is substantially influenced by the higher interlayer spacing of the organoclays^38^. It is also clear that the presences of MMT-A clay in the PP nanocomposite fabric improve the K/S value compared to those of either MMT-B or MMT-C using model disperse dye. The high K/S value obtained with MMT-A clay (double modification of MMT) may be due to its higher interlayer spacing than those of either MMT-B clay or MMT-C clay (single modification of MMT). Overall, SC-CO_2_ dyeing behavior of disperse dyes on the synthetic nanocomosite fabrics is an important issue.

### Effect of SC-CO_2_ dyeing pressure

**Figure 5:**
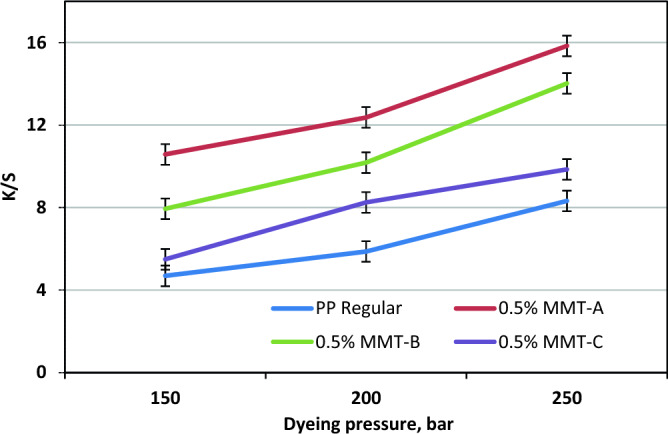
Effect of SC-CO_2_ dyeing pressure on K/S values of PP nanocomposite fabrics and their regular fabric at 120 °C, 1 h and 3% shade.

To study the effect of pressure on K/S values, experiments were conducted at pressure of 150, 200, and 250 bar at 120 °C, 3% shade and 1 h. Figure 5 demonstrates that K/S values of PP nancomposite fabrics and their regular fabric gradually increase with raising pressure. At lower pressure, the solubility of the disperse dye molecules is low, due to the relatively low densities of SC-CO_2_, which hindering the diffusion of disperse dye molecules towards the PP structures. Moreover, increasing pressure increases the density of SC-CO_2_ and also accelerating the solubilities of disperse dye molecules within PP structures. Consequently, the disperse dye molecules can easily diffuse into the PP structures, resulting in increased K/S values.

### Effect of SC-CO_2_ dyeing temperature

**Figure 6:**
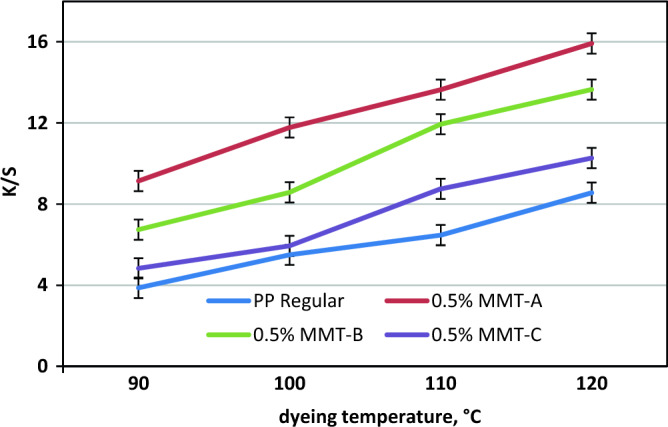
Effect of SC-CO_2_ dyeing temperature on K/S values of PP nanocomposite fabrics and their regular fabric at 250 bar, 1 h and 3% shade.

Figure 6 describes the effect of temperature on K/S values of PP nancomposite fabrics and their regular fabric dyed with model disperse dye in CO_2_ medium. Sequences of SC-CO_2_ dyeing practices were conducted to investigate the effect of dyeing temperature on K/S values at 250 bar, 3% shade and the dyeing time for 1 h. The K/S values are greatly influenced by the dyeing temperature and show an increase as the dyeing temperature increases from 90 to 120 °C. The lowest K/S values were found at 90 °C while the highest K/S values were obtained at 120 °C. These results suggest that higher dyeing temperature enhances the liberty of the macromolecular chains of PP structures, strengthening the adsorption of the disperse dye molecules towards the PP samples. Also, higher dyeing temperature accelerates the solubilities of disperse dye molecules and facilitates their penetration into PP samples, resulting in increased K/S values.

### Comparison of the color strength of SC-CO_2_ and aqueous dyeing method.

**Figure 7:**
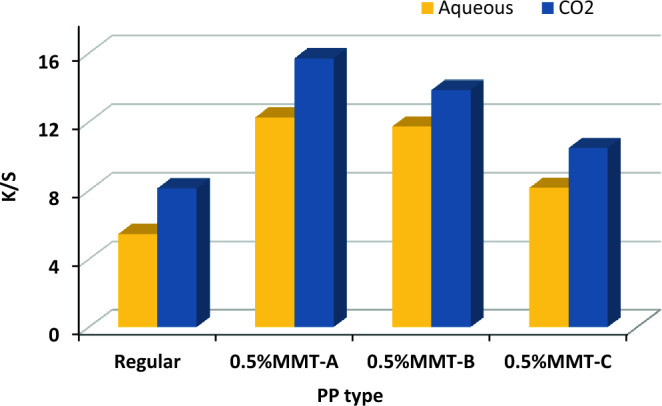
Comparison of K/S values of PP samples dyed in SC-CO_2_ medium at (250 bar, 120 °C, 3% shade and 1 h) and in aqueous medium at (3% shade, 120 ^o^C and 1 h).

Figure 7 describes the comparison between K/S values of PP fabrics dyed in SC-CO_2_ medium at (250 bar, 120 °C, 3% shade and 1 h) and those of samples dyed in aqueous medium at (3% shade, 120 °C and 1 h) using model disperse dye. The results indicated that, the dyed PP fabrics in SC-CO_2_ medium have higher K/S values than those dyed in aqueous medium. This is attributed to the fact that, dyeing in SC-CO_2_ medium enhances the liberty of the macromolecular chains of PP fabrics and improves the diffusion of disperse dyes into PP fabrics. This is in agreement with our previous wok^25^.

### Color coordinates of PP nanocomposite fabrics and their regular fabric dyed in SC-CO_2_ and aqueous dyeing media.

**Figure 8:**
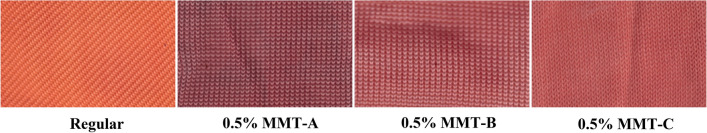
Images of dyed PP nanocomposite fabrics and their regular fabric in SC-CO_2_ medium at 250 bar, 120 °C, 3% shade and 1 h.

Table 2 shows the values obtained for the color coordinates (L*, a*, b*, C* and *h*°) of dyed PP fabrics in SC-CO_2_ medium at (250 bar, 120 °C, 3% shade and 1 h) and aqueous medium at (3% shade, 120 °C and 1 h) with model disperse red dye. It is clear that the dyed PP fabrics in SC-CO_2_ medium exhibited higher dye uptake than those dyed in aqueous medium, as evidenced by a decrease in L* values. The maximum L* values were found in the case of the PP regular fabrics, indicating a lighter shade compared to PP nanocompsite fabrics. It was observed also that all the colour coordinates of dyed PP samples in SC-CO_2_ and aqueous medium were positive with respect to red/green a* and yellow/blue b* coordinates depending on the uptake disperse red dye used; therefore, all of them lie in the yellow–red quadrant of the colour space diagram. *h*° values range from 22.04 to 43.52, indicating the red colour of all dyed PP samples. The average colour differences (∆E, calculated from the CIE L*a*b* coordinates) of the dyed PP fabrics show very good levelling properties. The results also indicate that the levelling of the dyed PP fabrics in SC-CO_2_ medium is slightly better than those dyed in aqueous medium. Images of dyed PP nanocomposite fabrics and their regular fabric in SC-CO_2_ medium are illustrated in Fig. 8.

**Table 2:** Color data of PP nanocomposite fabrics and their regular fabric dyed in SC-CO_2_ and aqueous media.

Dyed PP samples	Dyeing method	L*	a*	b*	C*	*h*°	∆E
Regular	SC-CO_2_	51.65	43.42	39.31	59.47	41.98	0.66
	Aqueous	56.65	36.80	36.98	41.49	43.52	0.73
0.5% MMT-A	SC-CO_2_	38.89	26.85	23.15	33.19	35.88,	0.56
	Aqueous	48.72	27.58	27.41	43.75	22.04	0.68
0.5% MMT-B	SC-CO_2_	43.58	28.44	20.82	33.95	36.15	0.58
	Aqueous	50.64	48.53	32.86	46.82	25.08	0.69
0.5% MMT-C	SC-CO_2_	46.73	34.78	25.64	42.16	35.71	0.59
	Aqueous	52.78	42.61	31.75	47.39	26.47	0.72

### Antibacterial properties of PP nanocomposite fabrics and their regular fabric dyed in SC-CO_2_ and aqueous dyeing media

The antibacterial behaviour of PP fabrics dyed in SC-CO_2_ medium at (250 bar, 120 °C, 3% shade and 1 h) and aqueous medium at (3% shade, 120 °C and 1 h) with model disperse red dye was evaluated against two bacterial strains; gram-negative *E. coli* (G −) and gram-positive *S. aureus* (G +) using *Colony Counting Method*. The results obtained are listed in Table 3. From which, the dyed nanocomposite fabrics exhibited higher antibacterial properties than those of regular fabrics which may be attributed to the action of the quaternary ammonium group/ long alkyl chain system in the silicate layer of the organo-modified clay used^39–41^. The antimicrobial activity of the quaternary ammonium group/long alkyl chain system in the organoclay could be due to the interaction with the predominantly anionic molecules at the cell surface, resulting in death of the cell^42–44^. The performance of the antibacterial properties against G + bacteria was found to be higher than that of G − which may be attributed to the difference in the structure of the two types of bacteria.

**Table 3:** Antibacterial properties of PP nanocomposite fabrics and their regular fabric dyed in SC-CO_2_ and aqueous media.

Dyed PP samples	Dyeing method	Bacterial reduction (%R)	
		G^−^	G^+^
Regular	SC-CO_2_	ND	12.86
	Aqueous	ND	12.14
0.5% MMT-A	SC-CO_2_	11.93	21.56
	Aqueous	11.27	21.12
0.5% MMT-B	SC-CO_2_	10.47	20.53,
	Aqueous	10.05	20.14
0.5% MMT-C	SC-CO_2_	9.76	19.72
	Aqueous	9.43	19.43

### Fastness properties of PP nanocomposite fabrics and their regular fabric dyed in SC-CO_2_ and aqueous dyeing media

Table 4 shows data for colour fastness to washing, perspiration and light for PP fabrics dyed in SC-CO_2_ medium at (250 bar, 120 °C, 3% shade and 1 h) and aqueous medium at (3% shade, 120 °C and 1 h) with model disperse red dye. As listed in Table 3, PP samples dyed in SC-CO_2_ and aqueous medium present excellent to very good washing, perspiration and light fastness.

**Table 4:** Fastness properties of PP anocomposite fabrics and PP regular fabric dyed in SC-CO_2_ and aqueous medium^a^.

Dyed PP samples	Dyeing method	Washing fastness*			Perspiration fastness*						Light
					Acidic			Alkaline			
		Alt	SC	SP	Alt	SC	SP	Alt	SC	SP	
Regular	SC-CO_2_	4–5	4	4	4–5	4	4	4–5	4	4	4–5
	Aqueous	4–5	4	4	4–5	4	4	4–5	4	4	4–5
0.5% MMT-A	SC-CO_2_	4–5	4	4	4–5	4	4	4–5	4	4	4–5
	Aqueous	4–5	4	4	4–5	4	4	4–5	4	4	4–5
0.5% MMT-B	SC-CO_2_	4–5	4	4	4–5	4	3–4	4–5	4	3–4	4–5
	Aqueous	4–5	4	4	4–5	4	4	4–5	4	4	4–5
0.5% MMT-C	SC-CO_2_	4–5	4	3–4	4–5	4	3–4	4–5	4	3–4	4–5
	Aqueous	4–5	4	3–4	4–5	4	3–4	4–5	4	3–4	4–5

*Alt, color change; SC, staining on cotton; SP, staining on polyester.

## Conclusion

PP nanocomposite fabrics and their regular fabric were dyed in SC-CO_2_ medium using model disperse red dye compared with aqueous medium. PP samples were dyed in SC-CO_2_ under the condition of different temperature and pressure. Results showed that the temperature and pressure had a clear impact on the dyeing of PP nanocomposite fabrics in SC-CO_2._ The dyed PP nanocomposite fabrics in SC-CO_2_ medium exhibited higher K/S values compared with their regular fabrics. The PP fabrics dyed in SC-CO_2_ medium have higher K/S values than those dyed in aqueous medium. The colourfastness of all dyed PP fabrics exhibited excellent to very good results towards washing, perspiration and light. The PP nanocomposite fabrics dyed in SC-CO_2_ and aqueous medium exhibited higher antibacterial properties than their regular fabric.

Using of SC-CO_2_ medium in dyeing PP nanocomposite fabrics achieved an obvious impact in enhancing their dyeing behaviour. A further study of optimum modified nanoclay structures would be of value for preparation of synthetic nanocomposite fabrics and their dyeing behaviour in SC-CO_2_ medium using selected disperse dye structures, and outcome of this study will be published in another publication.

## Data Availability

All data generated or analyzed during this study are included in this published article.
